# Impact of the 2018 ASCO/CAP guidelines on HER2 fluorescence in situ hybridization interpretation in invasive breast cancers with immunohistochemically equivocal results

**DOI:** 10.1038/s41598-019-53003-w

**Published:** 2019-11-13

**Authors:** Bo Wang, Wei Ding, Ke Sun, Xiaoling Wang, Liming Xu, Xiaodong Teng

**Affiliations:** Department of Pathology, the first affiliated hospital of Zhejiang University, Qingchun Road 79#, Hangzhou, China

**Keywords:** Breast cancer, Tumour biomarkers

## Abstract

The American Society of Clinical Oncology (ASCO)/College of American Pathologists (CAP) recently issued updated guidelines on human epidermal growth factor receptor 2 (HER2) testing by fluorescence *in situ* hybridization (FISH) in invasive breast cancers. In this study, we aimed to investigate the impact of the new recommendations on HER2 FISH interpretation in invasive breast cancers with immunohistochemically (IHC) equivocal results. 1810 breast cancer cases with IHC equivocal results were enrolled in this study between January 2012 and May 2019. Concomitant IHC was performed on the same tissue blocks detected by FISH testing. According to the 2018 guidelines, all the cases in ISH group 2 were categorized as HER2 negative; three of four cases in ISH group 3 were considered as HER2 positive, while the one scored IHC 1+ was reclassified as HER2 negative; Fifty-three previously ISH equivocal cases were redistributed into ten HER2-positive cases and forty-three HER2-negative cases. In conclusion, the utility of 2018 ASCO/CAP guidelines resulted in a slight decrease in HER2 positive rate, due to the reclassification of cases in ISH group 2 and group 4. The implementation of the new guidelines can reduce reflex FISH test and make the diagnosis of HER2 gene status more definitive.

## Introduction

Breast cancer is the most common carcinoma and the second leading cause of cancer-related death among Chinese women^1^. Amplification and/or overexpression of human epidermal growth factor receptor 2 (HER2) is observed in about 15–20% of invasive breast cancer patients^2^. HER2 positivity is closely related to the poor prognosis of patients^3–5^. Clinical trials have demonstrated that breast cancer patients with HER2 overexpression can benefit from the targeted therapy, showing the progression-free survival and the overall survival improvement^6–8^. Therefore, accurate assessment of HER2 status is a prerequisite for identifying the subset of breast cancer patients who may benefit from the anti-HER2 targeted therapy. Immunohistochemistry (IHC) and fluorescence *in situ* hybridization (FISH) are the two most frequently performed technologies to determine HER2 status.

The American Society of Clinical Oncology (ASCO)/College of American Pathologists (CAP) have periodically issued and updated the HER2 testing guidelines in breast cancers, with first version released in 2007, first revised in 2013 and focused updated in 2018. The new guidelines have addressed five clinical questions, including the following: (1) What is the most appropriate definition for IHC 2+ (IHC equivocal)? (2) Must HER2 testing be repeated on a surgical specimen if initially negative test on core biopsy? (3) Should invasive cancers with a HER2/chromosome enumeration probe (CEP17) ratio of ≥2.0 but an average HER2 copy number of <4.0 signals per cell be considered ISH positive (designated as ISH group 2)? (4) Should invasive cancers with an average HER2 copy number of ≥6.0 signals per cell but a HER2/CEP17 ratio of <2.0 be considered ISH positive (designated as ISH group 3)? (5) What is the appropriate diagnostic work –up for invasive cancers with an average HER2 copy number of ≥4.0 but <6.0 signals per cell and a HER2/CEP17 ratio of <2.0 and initially deemed to have an equivocal HER2 ISH test result (designated as ISH group 4)^9^? The diagnostic approach incorporates concomitant IHC review into ISH interpretation in ISH groups 2 to 4 to reach the most definitive HER2 status classification.

In this study, we retrospectively reviewed HER2 status in 1810 breast cancer patients with equivocal IHC results to assess the impact of these revised guidelines.

## Materials and Methods

### Case cohort

HER2 FISH results were collected from 1810 invasive breast cancer cases between January 2012 and May 2019. All cases were equivocal for HER2 IHC. Specimens included core needle biopsies, surgical excisions, and biopsy samples from metastatic sites. This research was approved by the Ethics Committee of the First Affiliated Hospital, College of Medicine, Zhejiang University. The Committee waived the written informed consents for the data were analyzed anonymously. All experiments were performed in accordance with relevant guidelines and regulations.

### IHC

Automated IHC for HER2 (Rabbit, clone 4B5; Ventana Medical Systems, Tucson, AZ) was performed on 4-um-thick tissue sections using an automated slide stainer, the Ventana Benchmark XT (Ventana Medical Systems). The results were interpreted according to the 2018 guidelines.

### Fish

4-um-thick tissue sections were deparaffinized, rehydrated, and immersed in distilled water for 40 min at above 90 °C. The slides were incubated for 18 min in protease solution at 37 °C. After dehydration with alcohol, a total of 10 uL HER2/CEP17 mixture probe (Jinpujia, Beijing, China) was added to the slides. The slides were then transferred to a hybridization oven (S500-24, Abbott molecular, USA). The procedure was as follows: denature at 83 °C for 5 min, and hybridization overnight at 42 °C. The second day, the slides were washed in preheated post-hybridization buffer, air dried and then counterstained with 15 μl DAPI. HER2 FISH signals were interpreted by one technologist (BW) and one pathologist (KS). Thirty nuclei from two non-overlapping areas were counted. When there was a conflict between the scores, another pathologist (XLW) would review the slide and reach the final result. The new guidelines were applied for the interpretation of FISH testing results.

## Results

A total of 1810 cases were enrolled in our study. Most of the cases (n = 1478) were sampled from surgical excisions. Specimens from core needle biopsies (n = 139) and metastatic sites (n = 72) were much less common (Table 1). According to the 2013 guidelines^10^, 318 cases with a HER2/CEP17 ratio ≥2.0 and ≥4.0 HER2 signals per cell (ISH group 1) were diagnosed as HER2 positive. 1406 cases with a HER2/CEP17 ratio <2.0 and <4.0 HER2 signals per cell (ISH group 5) were negative for HER2. There were 29, 4 and 53 cases in ISH group 2, 3 and 4 respectively. The positive rate was 19.40% (351/1810). Clinicopathological characteristics of the patients in each ISH group were summarized in Table 2.

**Table 1:** Distribution of cases from different sample types in the cohort.

	CNB(N)	SE(N)	MS(N)	Total
ISH group 1	25	221	12	258^a^
ISH group 2	3	25	1	29
ISH group 3	1	2	1	4
ISH group 4	14	35	4	53
ISH group 5	96	1195	54	1345^b^

N: number of cases; ISH: *in situ* hybridization; CNB: core needle biopsy; SE: surgical excision; MS: metastatic site.

^a^60 cases with missing data on the tumor characteristics excluded. ^b^61 cases with missing data on the tumor characteristics excluded.

**Table 2:** Clinicopathological characteristics of patients in each ISH group.

Characteristics	Value				
	ISH group 1	ISH group 2	ISH group 3	ISH group 4	ISH group 5
**Age**					
>60	38	5	0	11	363
≤60	220	24	4	42	982
NA^a^	60	0	0	0	61
**Tumor types**					
IDC	252	28	4	50	1262
ILC	1	0	0	1	29
Special types	5	1	0	2	54
NA^a^	60	0	0	0	61
**Tumor size**					
>2 cm	127	15	1	20	460
≤2 cm	94	10	1	15	735
NA^b^	97	4	2	18	211
**LN status**					
Positive	106	11	1	16	411
Negative	115	14	1	19	784
NA^b^	97	4	2	18	211
**WHO grade**					
I	4	0	0	0	191
II	87	12	0	13	572
III	124	13	2	20	354
NA^b^	103	4	2	20	289
**Hormone status**					
ER+	149	20	3	44	1152
ER−	109	9	1	9	193
PR+	130	16	3	38	1052
PR−	128	13	1	15	293
NA^a^	60	0	0	0	61

ISH: *in situ* hybridization; IDC: invasive ductal carcinoma; ILC: invasive lobular carcinoma;. LN: lymph node; WHO: world health organization; ER: estrogen receptor; PR: progesterone receptor.

NA^a^: not available for cases with missing data on the tumor characteristics. NA^b^: not available for cases with missing data on the tumor characteristics and not applied for samples from core needle biopsies or metastatic sites.

### Reclassification of cases in ISH group 2

Twenty-nine (1.60%) cases fell into ISH group 2. Among them, twenty-five cases were sampled from surgical excisions, three from core needle biopsies, and one from lymph node metastasis (Table 1). Most of the cases (n = 22) were scored as IHC 0/1+, and the remaining (n = 7) were assessed as IHC 2+ (Table 3). After a repeat FISH reading in seven cases with IHC 2+, the results remained the same (Table 3). Based on the 2018 guidelines, all the cases in this group were categorized as HER2 negative.

**Table 3:** Reassessment of HER2 FISH results in ISH Group 2, 3 and 4 based on the 2018 ASCO/CAP guidelines.

	Cases (N)	IHC result (N)		Recounting FISH in cases scored IHC 2 + (N)	
				HER2 negative	HER2 positive
ISH group 2	29	0	8		
		1+	14		
		2+	7	7	0
		3+	0		
ISH grouop3	4	0	0		
		1+	1		
		2+	2	2	0
		3+	1		
ISH group 4	53	0	8		
		1+	21		
		2+	15	14	1*
		3+	9		

N: number of cases; IHC: immunohistochemistry; FISH: fluorescence *in situ* hybridization.

*The result was derived from repeat FISH testing using another tissue sample with a HER2/CEP17 ratio of 2.19 and an average of 8.03 HER2 signals per cell.

### Reclassification of cases in ISH group 3

There were only four (0.22%) cases in ISH group 3, which were composed of two specimens from surgical excisions, one from lymph node metastasis, and one from core needle biopsy (Table 1). Two cases were scored as IHC 2+, while the other two were interpreted as IHC 3+ and 1+ (Table 3). FISH results of IHC 2+ cases remained the same following recounting targeted FISH (Table 3). The case scored IHC 1+ was from lymph node metastasis. Another block from its surgical excision was applied for further IHC confirmation and was also negative for HER2 (IHC 1+). According to the 2018 guidelines, three of four cases were considered as HER2 positive. The one scored IHC 1+ was reclassified as HER2 negative.

### Reclassification of cases in ISH group 4

**Figure 1:**
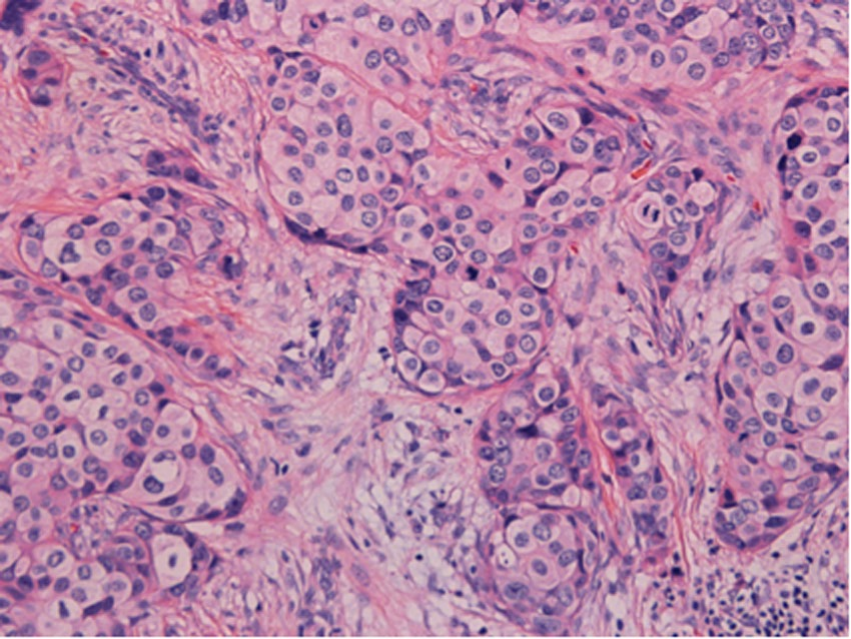
(Teng) (1 figure). Hematoxylin-eosin staining (HE) of a patient with invasive breast cancer in ISH group 4 (200X).

**Figure 2:**
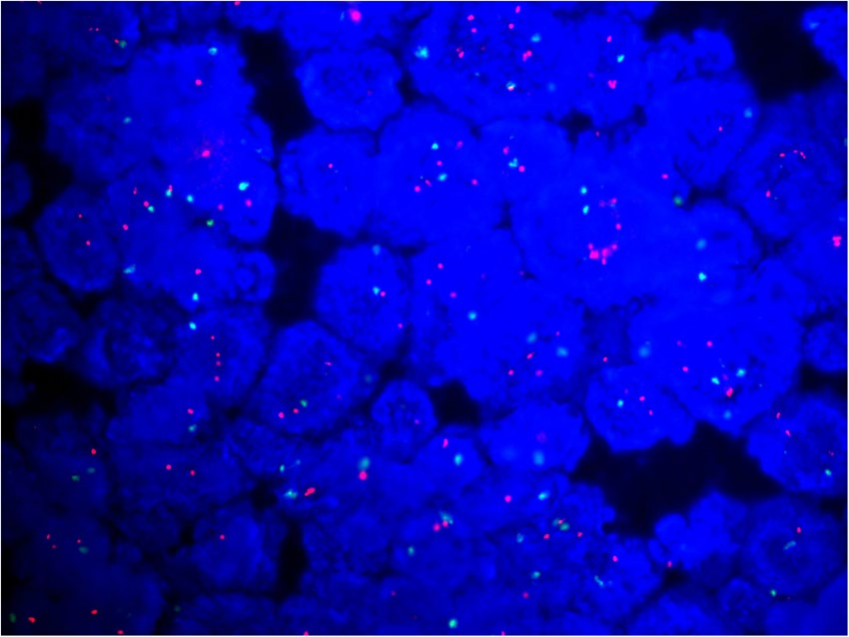
(Teng) (1 figure). The case was equivocal for HER2 FISH testing with a HER2 (red)/CEP17 (green) ratio of 1.8 and an average of 5.7 HER2 signals per cell.

**Figure 3:**
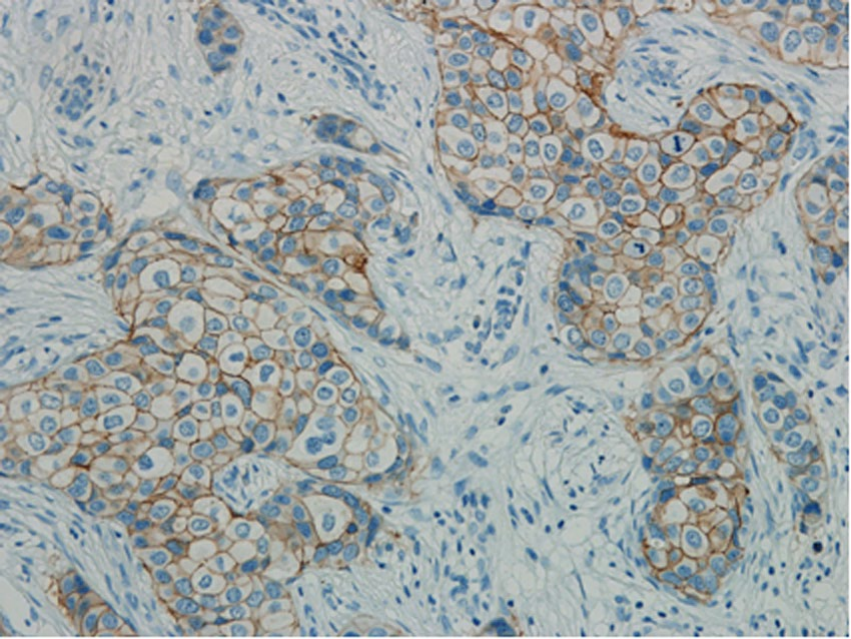
(Teng) (1 figure). Concomitant IHC was performed on the same tissue block used for FISH testing and it was scored as IHC 2+ (200X).

**Figure 4:**
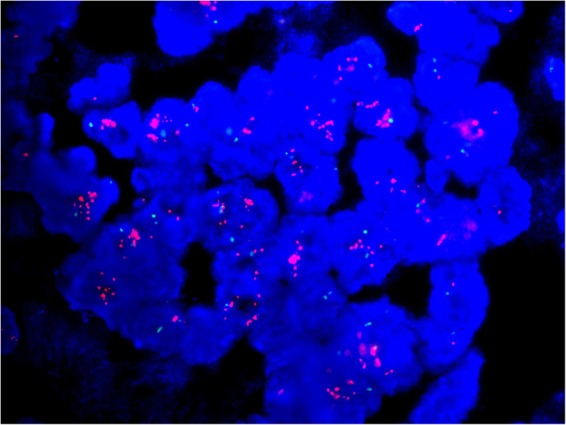
(Teng) (1 figure). Another tissue block was selected for further FISH testing in this case and it was turned out to be HER2 positive with a HER2 (red)/CEP17 (green) ratio of 2.19 and an average of 8.03 HER2 signals per cell.

Fifty-three (2.92%) of the 1810 cases fell into ISH group 4. Most of the cases (n = 35) were sampled from surgical excisions, followed by 14 from core needle biopsies and 4 from metastatic sites (Table 1). The number of cases scored as IHC 0, 1+, 2+ and 3+ was 8, 21, 15 and 9 respectively (Table 3). For IHC 2+ cases, reevaluation of FISH was performed. The counts remained a HER2/CEP17 ratio of <2.0 with an average of ≥4.0 and <6.0 HER2 signals per cell in all the cases. Another block for each case was collected for further FISH testing. Only one case was turned out to be HER2 positive with a HER2/CEP17 ratio of 2.19 and an average of 8.03 HER2 signals per cell (Fig. 1 (Teng), Fig. 2 (Teng), Fig. 3 (Teng), Fig. 4 (Teng)) (Table 3). After implementation of the 2018 guidelines, ten cases were converted to HER2 positive and forty-three cases were diagnosed as HER2 negative.

Taken together, 4.6% (83/1810) of the cases had a different final interpretation by 2018 guidelines compared with 2013 guidelines, including 30 previously considered HER2-positive cases being categorized as negative, 53 previously ISH equivocal cases being redistributed into 10 positive cases and 43 negative cases. In total, 331 of the 1810 cases were positive for HER2, with a slight decrease in positive rate from 19.4% to 18.3% (Table 4).

**Table 4:** Comparison of FISH results between 2013 and 2018 guidelines.

FISH status	2013 guidelines (N. %)	2018 guidelines (N. %)
Negative	1406 (77.68)	1479 (81.71)
Equivocal	53 (2.93)	0
Positive	351 (19.39)	331 (18.29)

N: number of cases; FISH: fluorescence *in situ* hybridization.

## Discussion

A good concordance rate between IHC and FISH test for HER2 in breast cancers has been reported^11–13^. As a result, in clinical practice, only those patients with IHC 2+ are referred for FISH analysis to determine the HER2 gene status in our institution. However, a small part of cases remain indeterminate for a HER2/CEP17 ratio of <2.0 with an average of ≥4.0 and <6.0 HER2 signals per cell which is defined as ISH equivocal in 2013 guidelines^10^. The aim of the 2018 recommendations is to update the guidelines to arrive at the most accurate designation of HER2.

The HER2 status in ISH group 2 is uncommon. Twenty-nine cases were included in this group, accounting for 1.60% of the present cohort (0.6% of all breast cancer cases), which was consistent with other reports^14–18^. Such cases were classified as HER2 positive according to the 2013 guidelines. Cases in this group were featured with low mean HER2 copy number and monosomy of chromosome 17^19^. Moreover, the clinical trials showed that trastuzumab therapy had no significant effect on the treatment of those patients in ISH group 2^9,14^. As a result, the 2018 guidelines recommend a definitive diagnosis be rendered based on additional work-up. Zare SY *et al*. studied 18 cases with a HER2/CEP17 ratio of ≥2.0 but an average HER2 copy number of <4.0 signals per cell and demonstrated that 11 cases were HER2 IHC negative, 7 were IHC 2+, and none of them were positive (3+)^20^. Another research showed that of 35 patients with this ISH pattern tested by IHC, only 3 cases were IHC 2+ and none of them were IHC 3+^14^. In parallel with these findings, our data also found that there was no case scored IHC 3+ in this group. So far as we know, only one case with HER2 IHC positive was reported in ISH group 2^21^.

There were only four cases in ISH group 3, with a lower incidence than other reports^15,17,21,22^. A ratio of <2.0 can be attributed to the increase in both HER2 and control centromere signals. All the four cases in the present study had gain of chromosome 17 (CEP17 ≥ 3.0). A remarkable variability of IHC score for cases in this group was observed across different laboratories^14,16–18,21^. The positive rate of IHC ranged from 8.3% to 75%. Cases with IHC 0/1+ could also be identified. Similarly, our data showed that two cases were scored as IHC 2+, while the other two were interpreted as IHC 3+ and 1+. Owing to the limited number of cases enrolled in clinical trials, a definitive conclusion on whether the patients with this ISH pattern can benefit from HER2-targeted therapy cannot be reached. Considering the heterogeneity of HER2 IHC results, the 2018 guidelines recommend that cases with concurrent IHC score of 2+/3+ be categorized as HER2 positive.

Fifty-three cases were assigned as ISH group 4. Before the introduction of the 2018 guidelines, the patients with equivocal ISH results posed a challenge to oncologists of whether to recommend HER2-targeted therapy. Moreover, implementation of the 2013 guidelines resulted in the detection of more equivocal cases^23–25^. Some laboratories relied on alternative chromosome 17 genes as surrogate to deal with this issue, leading to a change in HER2 status from equivocal to positive in about 50% of patients^15,26,27^. Due to the presence of false-positive HER2 results and the absence of evidence in the therapy efficacy, this approach has not been recommended in the 2018 guidelines. In our study, after concomitant IHC review, 29 cases were scored as IHC 0/1+, 15 were IHC 2+ and 9 were IHC 3+. Previous studies proved that HER2 genetic heterogeneity in breast cancers was most frequent in cases with IHC 2+ and equivocal HER2 amplification^28,29^. Therefore, another tissue block was selected for further FISH testing in the 15 cases with IHC 2+. Only one case was turned out to be HER2 positive with a HER2/CEP17 ratio of 2.19 and an average of 8.03 HER2 signals per cell. Finally, ten cases were converted to HER2 positive and forty-three cases were diagnosed as HER2 negative. The suggestion that repeat testing on the other tissue samples from the same patients in the new guidelines is appropriate in this setting.

In conclusion, the utility of 2018 guidelines resulted in a mild decrease in HER2 positive rate, due to the reclassification of cases in ISH group 2 and group 4. The implementation of the new guidelines can reduce reflex FISH test and make the diagnosis of HER2 gene status more definitive.

## Data Availability

The datasets generated and analyzed during the current study are available from the corresponding author on reasonable request.

## References

[1] Jemal A (2011). Global Cancer Statistics. CA Cancer J Clin.

[2] Wolff AC (2007). American Society of Clinical Oncology/College of American Pathologists guideline recommendations for human epidermal growth factor receptor 2 testing in breast cancer. Arch Pathol Lab Med.

[3] Kaptain S, Tan LK, Chen B (2001). Her-2/neu and breast cancer. Diagn Mol Pathol.

[4] Bundred NJ (2001). Prognostic and predictive factors in breast cancer. Cancer Treat Rev.

[5] Curigliano G (2009). Clinical relevance of HER2 overexpression/amplification in patients with small tumor size and node-negative breast cancer. J Clin Oncol.

[6] Smith I (2007). 2-Year follow-up of trastuzumab after adjuvant chemotherapy in HER2-positive breast cancer: a randomised controlled trial. Lancet.

[7] Schoffski P (2018). A phase Ib study of pictilisib (GDC-0941) in combination with paclitaxel, with and without bevacizumab or trastuzumab, and with letrozole in advanced breast cancer. Breast Cancer Res.

[8] Urruticoechea A (2017). Randomized Phase III Trial of Trastuzumab Plus Capecitabine With or Without Pertuzumab in Patients With Human Epidermal Growth Factor Receptor 2-Positive Metastatic Breast Cancer Who Experienced Disease Progression During or After Trastuzumab-Based Therapy. J Clin Oncol.

[9] Wolff AC (2018). Human epidermal growth factor receptor 2 testing in breast cancer: American Society of Clinical Oncology/College of American Pathologists clinical practice guideline focused update. J Clin Oncol.

[10] Wolff AC (2013). Recommendations for human epidermal growth factor receptor 2 testing in breast cancer: American Society of Clinical Oncology/College of American Pathologists clinical practice guideline update. J Clin Oncol.

[11] Vincent SA (2003). Calibration of immunohistochemistry for assessment of HER2 in breast cancer: results of the French multicentre GEFPICS study. Histopathology.

[12] Arnould L (2012). Accuracy of HER2 status determination on breast core-needle biopsies (immunohistochemistry, FISH, CISH and SISH vs FISH). Mod Pathol.

[13] Furrer D (2017). Concordance of HER2 immunohistochemistry and fluorescence *in situ* hybridization using tissue microarray in breast cancer. Anticancer Res.

[14] Press MF (2016). HER2 gene amplification testing by fluorescent *in situ* hybridization (FISH): Comparison of the ASCO-College of American Pathologists guidelines with FISH scores used for enrollment in breast cancer international research group clinical trials. J Clin Oncol.

[15] Shah MV (2016). Change in pattern of HER2 fluorescent *in situ* hybridization (FISH) results in breast cancers submitted for FISH testing: Experience of a reference laboratory using US Food and Drug Administration criteria and American Society of Clinical Oncology and College of American Pathologists guidelines. J Clin Oncol.

[16] Press MF (2016). Assessing the new American Society of Clinical Oncology/College of American Pathologists guidelines for HER2 testing by fluorescence *in situ* hybridization: Experience of an academic consultation practice. Arch Pathol Lab Med.

[17] Stoss OC (2015). Impact of updated HER2 testing guidelines in breast cancer–re-evaluation of HERA trial fluorescence *in situ* hybridization data. Mod Pathol.

[18] Ballard M (2017). ‘Nonclassical’ HER2 FISH results in breast cancer: Amultiinstitutional study. Mod Pathol.

[19] Page DB (2018). Monosomy 17 in potentially curable HER2-amplified breast cancer: prognostic and predictive impact. Breast Cancer Res Treat.

[20] Zare SY (2019). Breast cancers with a HER2/CEP17 ratio of 2.0 or greater and an average HER2 copy number of less than 4.0 per cell: frequency, immunohistochemical correlation, and clinicopathological features. Hum Pathol.

[21] Lin L, Sirohi D, Coleman JF, Gulbahce E (2019). American Society of Clinical Oncology/College of American Pathologists 2018 Focused Update of Breast Cancer HER2 FISH Testing Guidelines. Am J Clin Pathol.

[22] Liu ZH (2019). Impact of the updated 2018 ASCO/CAP guidelines on HER2 FISH testing in invasive breast cancer: a retrospective study of HER2 fish results of 2233 cases. Breast Cancer Res Treat.

[23] Long TH (2015). The New Equivocal: changes to HER2 FISH Results When Applying the 2013 ASCO/CAP Guidelines. Am J Clin Pathol.

[24] Muller KE, Marotti JD, Memoli VA, Wells WA, Tafe LJ (2015). Impact of the 2013 ASCO/CAP HER2 guideline updates at an Academic Medical Center that performs primary HER2 FISH testing: increase in equivocal results and utility of reflex immunohistochemistry. Am J Clin Pathol.

[25] Fan YS (2016). HER2 FISH classification of equivocal HER2 IHC breast cancers with use of the 2013 ASCO/CAP practice guideline. Breast Cancer Res Treat.

[26] Tse CH (2011). Determining true HER2 gene status in breast cancers with polysomy by using alternative chromosome 17 reference genes: implications for anti-HER2 targeted therapy. J Clin Oncol.

[27] Sneige N, Hess KR, Multani AS, Gong Y, Ibrahim NK (2017). Prognostic significance of equivocal Human Epidermal Growth Factor Receptor 2 results and clinical utility of alternative Chromosome 17 genes in patients with invasive breast cancer: A cohort study. Cancer.

[28] Ohlschlegel C, Zahel K, Kradolfer D, Hell M, Jochum W (2011). HER2 genetic heterogeneity in breast carcinoma. J Clin Pathol.

[29] Seol H (2012). Intratumoral heterogeneity of HER2 gene amplification in breast cancer: its clinicopathological significance. Mod Pathol.

